# A Systematic Review of Parents’ Knowledge of Children’s Oral Health

**DOI:** 10.7759/cureus.41485

**Published:** 2023-07-06

**Authors:** Manisha Kaushik, Shveta sood

**Affiliations:** 1 Pediatric and Preventive Dentistry, Manav Rachna Dental College, Faridabad, IND

**Keywords:** dental health, parents attitude, oral health, knowledge, children

## Abstract

Young children's oral health is maintained mainly by adults' knowledge and attitude. This study evaluated parents' attitudes, actions, and knowledge regarding their children's dental health. We searched the electronic MEDLINE, Embase, Cochrane, and PubMed databases. Additionally, each relevant article's and book's bibliography was thoroughly searched. Included were the phrases "Knowledge" [MeSH] ", Attitude" [MeSH] ", Parents" [MeSH] ", Children" [MeSH] "And Oral Health" [MeSH]. This review emphasizes the growing global interest in parents' contributions to children's dental health. It is necessary to raise awareness about the knowledge and significance of deciduous teeth, frequent dental appointments throughout society, and implement parental oral health education programs because parents need more awareness.

## Introduction and background

A vital part of good overall health has a healthy mouth. Even though having healthy teeth is only one aspect of having good oral health, many kids lack adequate dental and general health due to active and unchecked caries [1]. Oral health reflects overall health and cannot be separated from it. Fluoride exposure has dramatically improved children's oral health over the past 50 years, yet dental caries is still a severe public health issue that disproportionately affects low-income and minority populations. The well-being of a kid and their family can be significantly impacted by chronic pain from rotting teeth. It hinders their capacity to learn, flourish, and develop since it causes disturbed sleep and makes it difficult for them to eat because of pain [2].

In India, there needs to be more knowledge regarding the oral health of preschoolers. On the dental health of preschoolers, there needs to be more data. The mean decayed missing filled teeth (DMFT) was 1.40 [3], according to National Oral Health Survey (NOHS) 2000 data, which is relatively high compared to industrialized nations in Europe, North America, and Australia [4]. Streptococcus mutans is typically regarded as the primary etiological agent of dental caries, a contagious infectious illness [5]. Studies utilizing phenotyping and genotyping techniques significantly incline toward the hypothesis that the mother is the child's main point of infection. By encouraging the early establishment of S. mutans in newborns' and toddlers' mouths, improper feeding techniques used by mothers and other caregivers raise the risk of developing early childhood caries in those children [5].

Even when they attend preschools or nurseries, children under five often spend most of their time with their parents and guardians, especially mothers. The "primary socialization" in these formative years is when the initial routines and habits of childhood are formed [4]. These include good eating practices and healthy lifestyle choices that have become household norms and are reliant on the wisdom and conduct of parents and older siblings. According to studies, parents' negative attitudes regarding their children's oral health and the occurrence of caries are related [6].

Since the early years are crucial to preschoolers' growth and development, oral health is vital to overall health. Their health is crucial, and they must be free of sickness. Children at this age cannot make their own decisions; most of their active time is spent in school or with their parents, which makes the parent's role crucial for maintaining their kids' oral health and cleanliness [7]. Parents' knowledge influences a child's future dental health in this area. Parental awareness and habits about oral hygiene and health directly influence a child's dental health. As a result, parents should be viewed as a social force capable of ensuring early children's prosperity since they have the potential to improve the general oral health of the community's next generation. Their dedication can enhance the amount of preventative dental care a child receives at home, and their positive outlook can raise the demand for professional dental services [8]. 

Parents can significantly impact preventing oral illnesses in children by being directly accountable for their children's dental health. Children's teeth are cleaned, good hygiene and eating habits are taught, and expert dental treatment is arranged [9]. The two oral self-care behaviors that are most frequently practiced are using dental floss and brushing teeth [10]. Children typically pick up good oral hygiene habits by studying adults' attitudes and behaviors and listening to what they say. Education for children starts long before they ever see the dentist. The classroom is ideal for learning material while incorporating healthy habits from home. Good dental health habits are primarily the responsibility of parents, teachers, and dentists. Children learn by observation, perception, and active participation simultaneously. The educational intervention assumes a communication relationship exists. 

Even though there is not enough research to provide a precise prevalence estimate, the number is still very high based on the information that is now available. The preschool child's oral health depends heavily on their parents' understanding and awareness of oral hygiene preservation and future healthy eating habits. Since it is a preventable disease and the child depends entirely on their parents regarding oral health, measuring the parent's oral health-related knowledge is very important. This study assessed parents' attitudes about and understanding of oral health practice.

## Review

Methods

This study followed the preferred reporting items for systematic review and meta-analyses statement.

Knowledge, attitude, and perception practice (KAP) studies related to the oral health of children published in peer review scientific journals from April 2009 to September 2021 with English as the publishing language, all the studies in which the outcome was illustrated in terms of KAP, all the articles in the English language as well as articles published within the period of 31 September 2021 were included in this study. Articles with language other than English and articles with incomplete patient data were excluded from this study.

Search Strategy 

We searched the electronic MEDLINE, Embase, Cochrane, and PubMed databases. Additionally, each relevant article's and book's bibliography was thoroughly searched. The pertinent papers were chosen by two reviewers separately based on the inclusion and exclusion criteria. The two reviewers debated any differences until they agreed.

Methodological medical subject heading (MeSH) phrases were created using the patient population, intervention, comparison, and outcome (PICO)-format inquiry to increase the sensitivity of the search approach for locating research. These terms included "Knowledge" [MeSH] and "Attitude" [MeSH] as well as "Parents" [MeSH], "Children" [MeSH], and "Oral Health" [MeSH]. Studies that satisfied these requirements were subjected to critical evaluation. The listed studies' merits were assessed using a suggested unique quality assessment scale.

Selection

Three steps were taken in the selection of the studies. First, all article titles were examined, and suitable studies were chosen per the inclusion and exclusion criteria. Abstracts for each of the chosen titles were acquired, examined, and relevant abstracts were chosen based on the criteria. Finally, the definitive collection of articles was obtained while keeping in mind the selection criteria after full-text versions of all the abstracts that had been chosen had been obtained and examined, and finally, six articles were selected for the study (Table 1).

**Table 1:** Selection of articles

Initial search	180
Duplicates and non relevant	71
Case reports and series	15
Reviews	62
Abstract	13
Language other than English	13

Data Extraction

The data extraction forms were used to extract the data. Authors, study year, study design, knowledge questions, and attitude questions were taken out of the data.

Quality Assessment

The quality of the studies was evaluated using the Cochrane collaboration tool for assessing the risk of bias in randomised controlled trials (RCTs) [reorder buffer (ROB) 2]. The Newcastle-Ottawa quality Assessment Form for Cohort Studies, the Oxford Systematic Review Appraisal Sheet, the Critical Appraisal Skills Programme, and the Grading of Recommendations Assessment Development and Evaluation (GRADE) system for grading evidence were all used to ensure the accuracy of the data analysis in this systematic review. The pursuing things were assessed: examples of selection bias include random sequence generation and allocation concealment, performance bias, attrition bias, reporting bias, and any other prejudice found. Each prejudice received a risk assessment of high, low, or unsure. Three observers independently assessed the circumstance, and any disagreements were discussed. The procedure for this systematic review was developed using accepted concepts. A clear review question was also developed using the PICO paradigm.

Results

**Figure 1:**
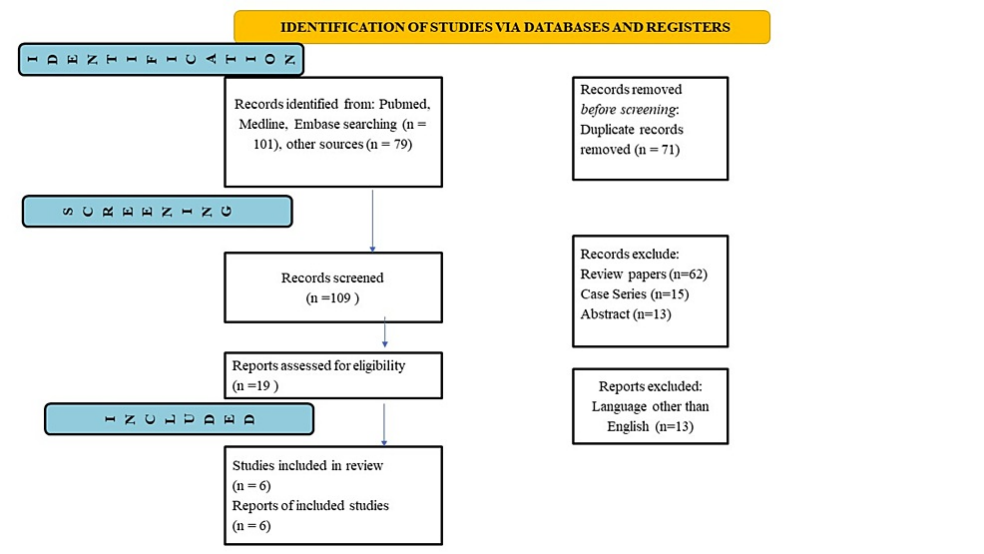
Prisma flowchart n= total number

Initial searches yielded 180 articles. Six studies were considered for analysis out of 180 articles found in the database after duplicates were removed and publications that did not meet eligibility requirements were eliminated. The PRISMA flowchart for the inclusion of studies is shown in (Figure 1).

Initial searches yielded 180 articles, out of which 101 articles were obtained from Medline, Pubmed, and Embase, while 79 were obtained from other sources. A total of 71 duplicate records were excluded before the screening. A total of 109 records were screened, of which 62 review papers, 15 case series, and 13 abstracts were excluded. Six studies were included in the report and reviewed. Narrative synthesis has been provided for the findings obtained from the studies. Three studies were questionnaire-based [11-13], while the other three were cross-sectional [14-16]. The data extracted has been presented in tabular form in Table 2, as mentioned below.

**Table 2:** Included studies

Author	Type of Study	Country	KAP
Bodhale P et al., [11]	Questionnaire based study	Nasik, India	Yes
Mounissamy A et al., [12]	Questionnaire based study	Chennai, India	Yes
Kumar G et al., [13]	Questionnaire based study	New Delhi, India	Yes
Almulhim B et al., [14]	Cross-sectional	Riyadh, Saudi Arabia	Yes
Khanduri N et al., [15]	Cross-sectional	Bhairahawa, Nepal	Yes
Alshammary F et al., [16]	Cross-sectional	Hail, Saudi Arabia	Yes

A total of six studies were included with positive responses, out of which four studies included questions regarding primary teeth importance and dental care with positive responses ranging from 50%-94.18% [12-14,16]. Two of the studies mentioned the questions about regular dental visits and the importance of fluoridated toothpaste [11,15]. The studies included in this systematic review with positive findings were tabulated (Table 3).

**Table 3:** Positive findings of the included studies

Author	Questions included	Positive response of parents
Bodhale P et al., [11]	When to start brushing primary teeth	50%
	Right timing of the first dental visit	43.1%
	Effect of dental health on general body health	63.8%
Mounissamy A et al., [12]	Regular visits to the dentist are important	55.7%
	Treating primary tooth is necessary	71.7%
	Using fluoridated toothpaste/powder	31%
Almulhim B et al., [14]	Primary teeth need dental care same as permanent teeth,	85.15%
	Less than 1 year age at which they start brushing their children’s teeth,	44.55%
	Fluoride prevents tooth decay	77.23%
Khanduri N et al., [15]	Importance of using fluoridated toothpaste	25%
	Importance of brushing teeth	70%
	Necessary to take the child for regular dental visits	75%
Kumar G et al., [13]	Perception of the importance of primary teeth	89%
	First dental visit after dental problem	84.6%
	Fluoridated toothpaste used	94.8%
Alshammary F et al., [16]	Do you think that primary teeth are important?	55.9
	Do you think that problems in primary teeth can affect the permanent teeth?	50.7%
	Effects of prolonged bottle-feeding on children’s oral health	49.78%

Risk of Bias Assessment

The risk of bias was evaluated using the Cochrane risk of bias assessment tool. Each element from one of five domains is given a biased score (high, low, or unclear) (selection, performance, attrition, reporting, and others). Part I of the Quality Assessment Form evaluates the risk of selection, reporting, and other biases. Using the Quality Assessment Form Part II, performance, detection, and attrition bias risk is evaluated. The risk of bias was classified for each judgment as "high," "low," or "unclear" using the instructions at the bottom of the questionnaire (Table 4).

**Table 4:** Risk of bias in the included studies

Authors name	Selection Bias Random sequence generation	Allocation Concealment	Reporting bias	Others	Performance bias Blinding participants and personnel	Blinding Outcome	Attrition bias
Bodhale P et al., [11]	Low risk	Low risk	Low risk	Low risk	Low risk	Unclear	High risk
Mounissamy A et al., [12]	Low risk	Low risk	Low risk	Low risk	Low risk	Low risk	Low risk
Almulhim B et al., [14]	Low risk	Low risk	Low risk	Low risk	Low risk	Low risk	Low risk
Khanduri N et al., [15]	Low risk	Unclear	Low risk	Low risk	Low risk	Unclear	Low risk
Kumar G et al., 2019 [13]	Low risk	Low risk	Low risk	Low risk	Unclear	Unclear	Low risk
Alshammary F et al., 2019 [16]	Low risk	Low risk	Low risk	Low risk	Low risk	Low risk	Low risk

Discussion

Since oral health-related habits (such as those connected to oral hygiene and diet) are acquired during infancy and maintained throughout early childhood, the children's oral health is correlated with the oral health knowledge of their mothers/guardians [17]. The research on parents' knowledge and practices in children's dental health has never been mapped and compiled. We aimed to locate all available papers without excluding any publications based on their quality or research design. Finding and synthesizing publications with a specific emphasis on quality assessment is the goal of a systematic review. The process of merging data from studies with a high level of evidence, such as randomized controlled trials is known as the meta-analysis [18]. 

Children's dental health mainly depends on their parents' awareness because early oral health habits are formed during infancy and maintained throughout early childhood [19]. Dental caries is a prevalent chronic infectious disease resulting from tooth-adherent cariogenic bacteria that metabolize sugars to produce acid, which over time demineralizes tooth structure. Dental caries is a disease that can be prevented. Parents can save precious time and money on dental care if preventive measures are implemented at a young age. Since the preschool age group (two to four years of age) depends on them for their oral healthcare needs, oral health education of parents is therefore crucial. Later, oral health promotion methods such as appropriate brushing and fluoridated toothpaste can be advocated in collaboration with the parents. In order to develop preventative strategies, it is necessary to evaluate the current levels of knowledge, attitude, and habits.

Most of the research included in this review was cross-sectional and assessed parents' knowledge and behavior through self-reported surveys-only two of the studies we examined used observational techniques. Self-reported practice surveys and knowledge tests are helpful, although practice self-reports may not match real practice. Studies that observe actual practice and audit customer files may help advance knowledge in this area.

Only a few of these studies included power calculations, and their sample sizes varied. Using established concepts to construct surveys might seem logical, but reaching a global agreement on a uniform tool would enable evidence pooling and intra- and inter-country comparisons.

Studies promoting oral health must understand social, economic, belief, behavioral, and attitudinal aspects. A central model based on oral health promotion initiatives (the 1970s-1980s) was created to give the populace comprehensive information and recommendations on oral health-related behaviors. A broader perspective, including several determinants of oral health, is required to encourage people from all origins to embrace a healthy lifestyle [20,21].

Part of the family-wide oral health practices is attributed to socioeconomic differences. Oral health inequalities are unlikely to be eliminated by oral health initiatives intended to alter habits [22-24]. Kay et al. in their opinion, share that health education aids in increasing knowledge and modifying attitudes and beliefs [25]. Health promotion initiatives give parents and pupils the necessary information about dental care, including oral health behaviors and attitudes. However, dental hygiene should be the responsibility of the entire family. Individual, family, and societal levels are addressed when addressing health problems [26-28].

 In actuality, individual-based simple models have their limitations and are no longer appropriate. Families are part of communities, and children live in families. Therefore, children's dental health is tied to successful community programs like public outreach and oral health promotion. Children with superior oral health live in communities emphasizing oral health [29]. General health and oral health are related. The mouth is a part of the body, and a child's risk of acquiring oral disorders is comparable to illnesses of the whole body [30-32].

 It is also impossible to distinguish between a child's risk of developing general and oral illnesses and their family and society's risk for disease. As a result, any strategy for improving children's oral health must be built on a multi-layered outlook to have a lasting impact [33]. It is essential to comprehend the concept behind motivating both individuals and communities. The most challenging patients to work with when providing oral health education are those with the lowest motivation and risk of developing caries [34]. There is evidence of risk-based referral in several of the studies. It might be appropriate if children's risk status is adequately assessed. However, the study data is needed to allow us to do so. In many instances, an existing, irreversible condition (such as cavitation) was the driving force behind the referral rather than a risk assessment. The value of time spent on dental health, as opposed to more commonplace and comfortable pursuits, may only be recognized if evidence-based therapies (and the extent of their benefit) are well understood or if compensation for oral health is negligible or nonexistent.

## Conclusions

Parents' awareness of their attitudes and knowledge of their children's oral health could be higher. Parents can significantly impact the development of healthy oral habits for their children by modeling healthy behaviors for them. There is a need to educate society about deciduous teeth, their importance, and the necessity of visiting the dentist regularly. The importance of teaching expecting and new mothers about baby oral health care, including the use of nursing bottles at night and routine dental visits. This study reveals a new facet of the crucial function played by the pedodontics triangle in planning parental oral health education initiatives. First, the public needs to be made more aware of the value and necessity of a first visit to the dentist. Because of this, oral health educators who run awareness campaigns in various settings and communities will find this study thought-provoking.

In conclusion, many parents had sound knowledge, but their attitudes and practices needed to match. Regular oral health promotion education programs are beneficial, focusing on parents' attitudes toward the treatment options available to their kids. This study also underlines the necessity for Indian society to adopt a positive attitude toward treatment options for primary teeth.
